# Nitrogen use aggravates bacterial diversity and network complexity responses to temperature

**DOI:** 10.1038/s41598-022-15536-5

**Published:** 2022-08-17

**Authors:** Xiaoyi Xing, Huifang Xu, Dou Wang, Xianjun Yang, Hongling Qin, Baoli Zhu

**Affiliations:** 1 Urban and Rural Construction College, Shaoyang University, Shaoyang, 422004 China; 2 Key Laboratory of Agro-Ecological Processes in Subtropical Regions, Taoyuan Agro-Ecosystem Research Station, Institute of Subtropical Agriculture, Chinese Academy of Sciences, Changsha, 410125 China; 3 Research Center on Ecological Sciences, Jiangxi Agricultural University, Nanchang, 330045 China; 4 Gansu Provincial Key Laboratory of Arid Land Crop Science, Agronomy College of Gansu Agricultural University, Lanzhou, 730070 Gansu China

**Keywords:** Community ecology, Bacteria

## Abstract

Rising temperature affects microbial composition and function in agriculture field, especially under nitrogen fertilization. In this study, we investigated the bacterial community of paddy soil incubated at controlled temperatures (5 °C, 15 °C, 25 °C, and 35 °C). Results showed that the response of bacterial communities to temperature was not uniform. Temperature elevation from 15 to 25 °C abruptly shifted the soil bacterial community, whereas elevation from 5 to 15 °C and from 25 to 35 °C had a marginal effect. The bacterial α-diversity was higher at 5 °C and 15 °C, owing to the massively distributed taxa with low abundance. However, as the temperature increased to 25 °C and 35 °C, these taxa were diminished, whereas *Firmicutes* significantly increased, resulting in a strong decline in α-diversity. Simultaneously, bacterial network complexity significantly increased at 25 °C and 35 °C, indicating the bacteria had closer interactions. Nitrogen application aggravated the variation in bacterial diversity and network complexity among temperatures. Interestingly, most complex network was observed under higher temperatures in fertilized soils. Collectively, these results indicate that nitrogen exacerbates the response of the soil bacterial community to temperature, and association between diversity and network complexity may be present.

## Introduction

Climate change, with its main feature of warming, profoundly affects various ecological systems on the planet^1^. The global mean surface temperature has risen by 0.78 °C since industrialization and is predicted to increase by 1.5–4.5 °C by the end of the twenty-first century^2^, based on mean values. Along with the increase in the mean temperature, extreme warming events have also intensified^3^, resulting in huge temperature increase in different seasons. For example, Cardoso et al.^3^ predicted a maximum of 8 °C increment in summer in the Mediterranean area. Over the past two decades, China also suffered from frequent warming events^4^. Both northwest and southern China experienced daily maximum temperature greater than 35 °C for several weeks in 2016^4^.

Microbial diversity is closely related to a variety of ecosystem processes and functions, such as soil nutrient cycling, and is associated with ecosystem stability and health^5–7^. Several studies have found that warming exerts strong effects on microbial diversity; however, results have not been consistent^8–12^. A midst of global warming, some regions have been cooling over recent decades, such as the Andes, Northern Greenland, and the Southern Ocean subsurface^13,14^. However, the impact of cooling on microbial diversity is not a widespread concern. Sabri et al.^15^ reported that the soil microbial diversity increases after soil is cooled at 4 °C. To date, we cannot determine the impact of the changing temperature on microbial diversity based on previous studies.

When the function of the ecosystem is damaged, owing to a change in diversity, microbial communities may functionally compensate to maintain key ecological processes^5,16^. For example, a decrease in microbial diversity would lead to changes in the abundance of certain species owing to changes in competitor and predator populations; this phenomenon is referred to as density compensation^16,17^. In addition, soil microbes that coexist in an ecosystem interact with each other, forming complex ecological webs^18^ that are important for ecological function^19,20^. Microbial interactions can be represented as networks, with nodes and links indicating the species and their relationships, respectively^21,22^. Several studies have shown that stronger microbial interactions, which are represented by more complex and closer networks, can improve nutrient cycling^19,20^. A mechanistic link exists between species interactions and biodiversity in microbial ecosystems^19,23^ because the interaction between microbes strongly influences the presence or absence of other organisms^24^. For example, Zhao et al.^25^ found that a lower microbial diversity is associated with more complex and closer interactions in subtropical and tropical forest soils. Additionally, laboratory experiments using microbial cultures have shown that increased microbial interactions lead to lower biodiversity^23^. Therefore, we aimed to know whether microbial diversity and interactions have an intrinsic relationship.

To date, the effect of temperature on potential microbial associations has rarely been studied as compared to microbial diversity and composition^26,27^. For instance, Wan et al.^26^ found that soil bacterial networks in paddy soil samples are mainly shaped by soil temperature. Yuan et al.^27^ reported that warming significantly increased the robustness and complexity of microbial network and maintained the functional potential of microbial community in grasslands. Since relatively few studies are available in this field, this phenomenon needs further clarification based on various ecosystems and fertilizer types. Whether and how elevated temperature impacts soil bacterial network complexity and its relationship with bacterial diversity remain to be uncertain.

The use of nitrogen (N) fertilizer is also one of the most frequent anthropogenic activities in agricultural ecosystems, increasing by a factor of nine from the 1960s to the present^28^. Many soil microbes participate in soil N turnover; therefore, N fertilization profoundly influences soil microbial communities. Several studies have suggested that N addition suppressed soil microbial diversity^11,29^, especially when the nitrogen content is high^30^. Furthermore, fertilization has been reported to simplify the complexity of the soil microbial community network^31,32^. In addition, N availability influenced the response of the soil microbial community to temperature^33–35^. However, these findings were based on soil respiration, which could only represent the living state of soil microbes to some extent. To our knowledge, no studies have examined how N affects soil microbial diversity and interspecies interactions under different temperatures.

As the largest consumer of N fertilizers in the world, China is facing widespread overuse in agricultural ecosystems^31,36,37^. The average rate of N use in paddy fields is less than 100 kg per hectare worldwide, whereas that in several Chinese provinces often exceeds 200 kg per hectare^31^. Therefore, we aimed to explore the influence of high N levels on bacterial diversity and networks under different temperatures, as well as the presence of compensation mechanism between them.

In the present study, we designed simulated incubations to investigate how temperature influences soil bacterial diversity and interactions under different N content in a subtropical zone and explore whether a compensatory mechanism exists between bacterial diversity and interspecies interactions. We hypothesized the following: (1) nitrogen availability would alter the response of soil bacterial diversity to temperature, and (2) bacterial interspecies interactions would change in response to changes in biodiversity. The results of this study may provide a deeper insight into the ecological consequences of excessive nitrogen fertilization during future climate change.

## Results

### Bacterial abundance

**Figure 1:**
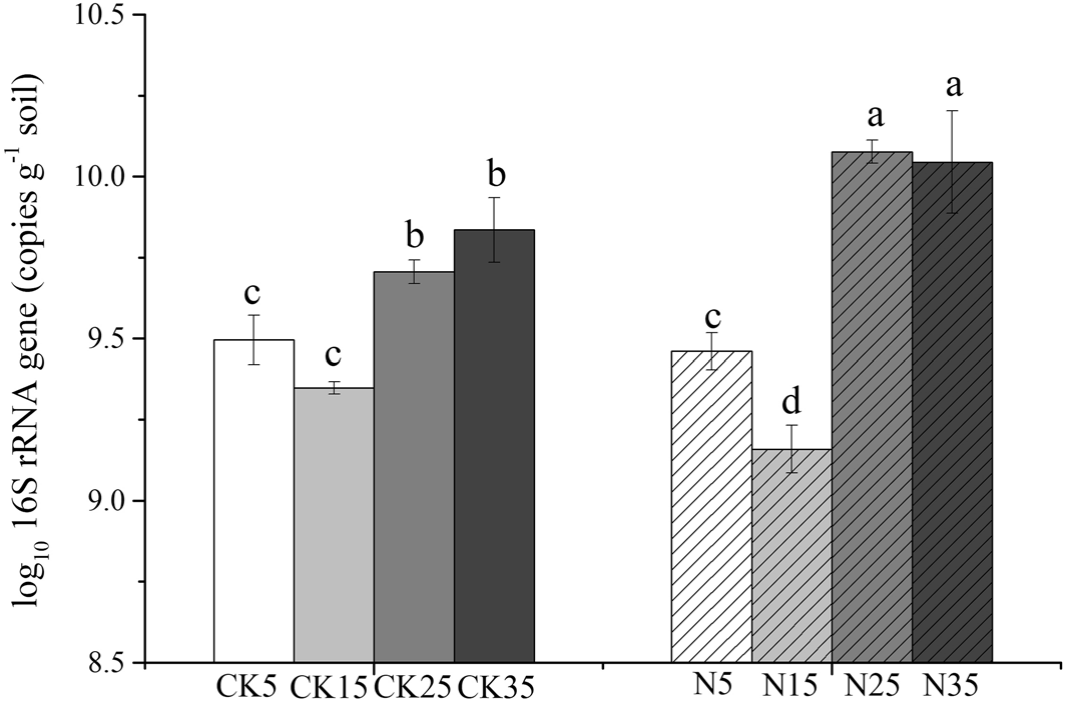
Soil bacterial abundance at different incubation temperatures. Data are presented as mean and standard error (n = 3). Data followed by different letters indicate significant differences (*P* < 0.05). CK, soil samples not treated with nitrogen; N, soil samples treated with nitrogen.

The total bacterial abundance varied between 1.27 × 10^9^ and 1.49 × 10^10^ copies of the 16S rRNA gene per gram of soil (Fig. 1). In general, elevated temperatures led to higher bacterial abundances, wherein the most significant variation was observed between 15 and 25 °C. Additionally, N fertilization exerted different effect on the bacterial abundance at different temperatures. Generally, N significantly increased bacterial abundance by 2.35 and 1.66 times their levels without N use at 25 °C and 35 °C, respectively. Contrarily, the effect of fertilization was lower at 5 °C and 15 °C.

### Bacterial α-diversity

The Ace, Chao1, Shannon, Simpson, and PD indices were calculated to explore the α-diversity of soil bacteria under different treatments. Results showed that α-diversity generally decreased with increasing incubation temperatures (Table 1), wherein a significant decrease was observed between 15 and 25 °C. Contrarily, the use of N application exerted different effects on bacterial α-diversity at low and high temperatures, with varying degrees (Table S1). In particular, at low temperatures, N slightly increased Ace, Chao1, Shannon, and PD indices and decreased the Simpson index compared to CK treatment. The mean Chao1 index slightly increased by 5%. Furthermore, N significantly decreased the diversity indices, except for the Simpson index, at 25 °C and 35 °C. For instance, Chao1 index was significantly decreased by approximately 25%. Thus, temperature and N fertilization had interactive effects on bacterial alpha diversity.

**Table 1:** Bacterial α-diversity under different incubation temperatures.

	Ace	Chao1	Shannon	Simpson	PD
CK5	2268 ± 76 ab	2254 ± 71 a	5.09 ± 0.04 b	0.048 ± 0.01 b	130 ± 3.40 b
CK15	2266 ± 49 ab	2214 ± 37 a	5.14 ± 0.06 b	0.040 ± 0.01 cd	129 ± 3.92 b
CK25	2084 ± 63 b	2039 ± 53 b	5.05 ± 0.22 b	0.036 ± 0.01 cd	118 ± 7.26 c
CK35	2026 ± 7 b	2006 ± 22 b	5.15 ± 0.12 b	0.029 ± 0.00 de	114 ± 3.41 c
N5	2380 ± 31 a	2363 ± 32 a	5.92 ± 0.06 a	0.019 ± 0.00 f.	150 ± 0.75 a
N15	2386 ± 19 a	2349 ± 43 a	5.86 ± 0.08 a	0.020 ± 0.00 ef	147 ± 2.36 a
N25	2066 ± 124 b	1609 ± 99 c	2.97 ± 0.26 c	0.158 ± 0.01 a	89 ± 9.07 d
N35	1887 ± 55 c	1428 ± 117 d	3.16 ± 0.15 c	0.130 ± 0.02 b	82 ± 2.48 d

All data are presented as mean ± standard deviation (n = 3).

CK, soil samples with no nitrogen application; N, soil samples with nitrogen application. Differences in bacterial alpha diversity among different treatments was analyzed using one-way ANOVA. Data followed by different letters indicate a significant difference (*P* < 0.05).

### Bacterial distribution

**Figure 2:**
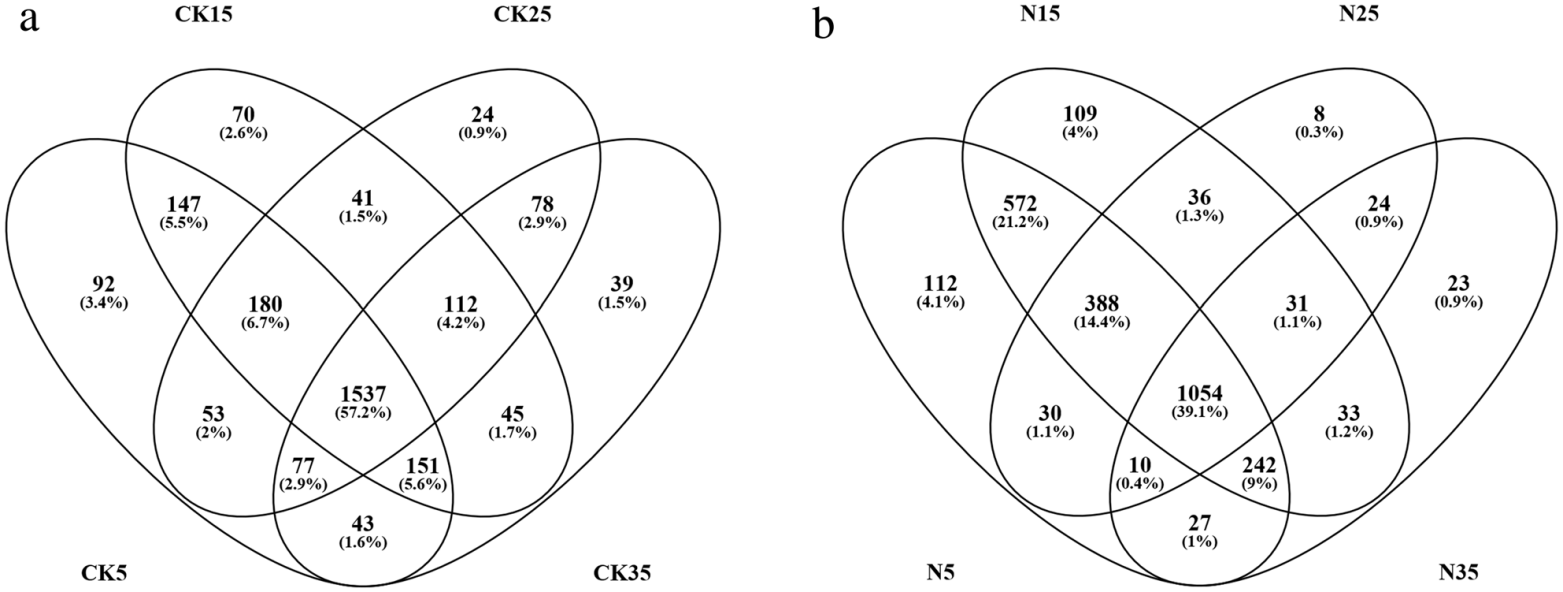
Venn diagram showing the shared soil bacteria OTUs at different conditions. CK, soil samples with no nitrogen application (**a**); N, soil samples with nitrogen application (**b**).

**Figure 3:**
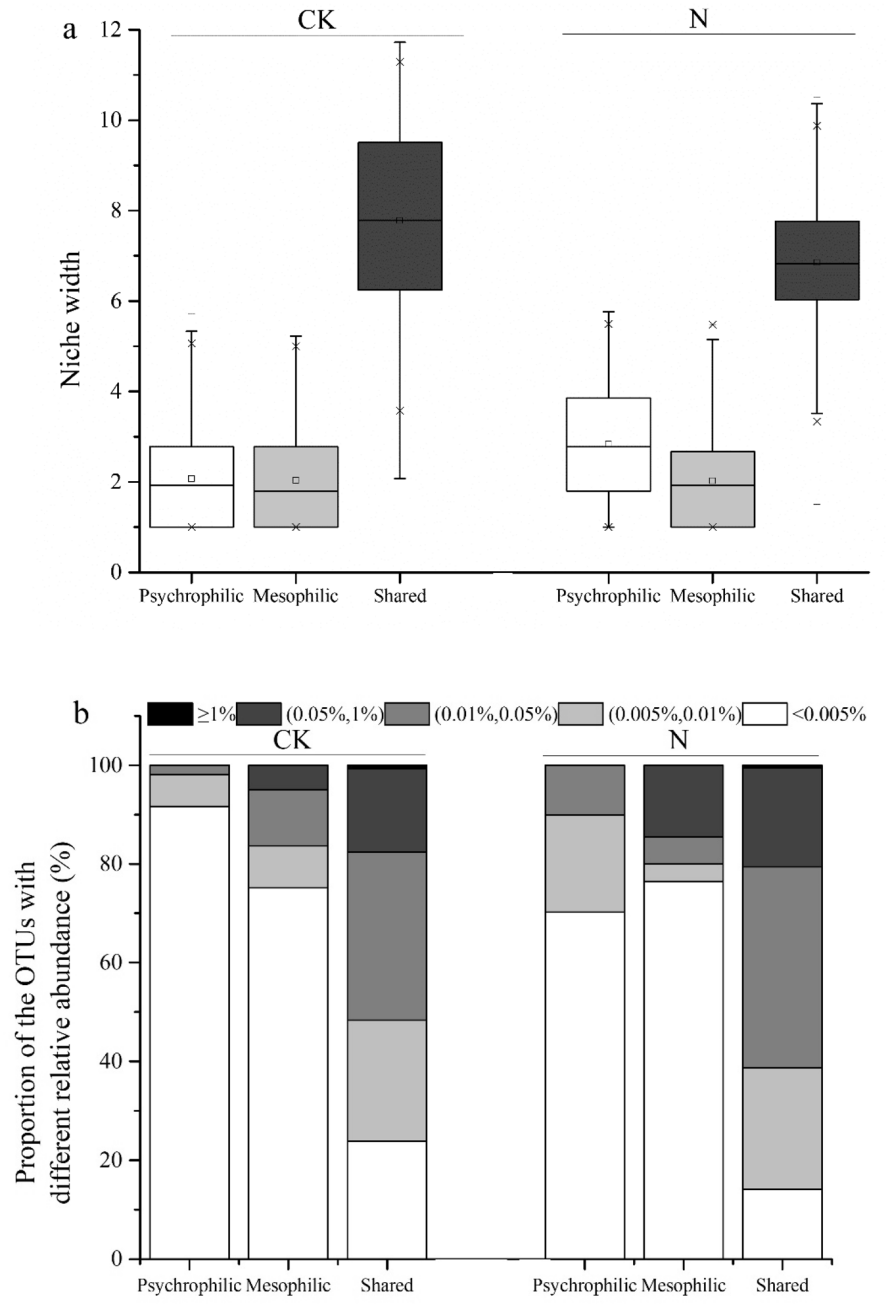
Niche width (**a**) and abundance frequency (**b**) of psychrophilic, mesophilic, and shared OTUs. CK, soil samples with no nitrogen application; N, soil samples with nitrogen application; psychrophilic OTUs, OTUs that are present at 5 °C and/or 15 °C treatments; mesophilic OTUs, OTUs that are present at 25 °C and/or 35 °C treatments; shared OTUs, OTUs detected in all treatments. In (**b**), ≥ 1% represents relative abundance higher than or equal to 1%, < 0.005% represents that lower than 0.005%, (0.05%,1%) represents relative abundance higher than or equal to 0.05% and lower than 1%, and so on.

A Venn diagram was constructed to explore the incidence of taxa in soils at high temperatures. We categorized the OTUs that were detected at 5 °C and 15 °C as psychrophilic OTUs, whereas those that are present at 25 °C and 35 °C were categorized as mesophilic OTUs. OTUs that were present in all treatments were named as shared OTUs. We identified 309 and 793 psychrophilic OTUs and 141 and 55 mesophilic OTUs in the control and fertilized soils, respectively (Fig. 2). Although a few new bacterial species emerged to compensate for biodiversity loss, many bacterial species were diminished, especially under combined fertilization and high temperature condition. Further analysis revealed that the mean Bcom (community-level habitat niche breadths) values of the psychrophilic and mesophilic OTUs were much lower than those of the OTUs shared by all communities (Fig. 3a). Moreover, the relative abundance of most psychrophilic OTUs was very low (< 0.005%); contrarily, some mesophilic OTUs had a higher relative abundance (> 0.01% or 0.05%) (Fig. 3b). These results suggest that most psychrophilic OTUs did not survive in their own narrow ecological niche and were removed under warming phenomena. Meanwhile, a few mesophilic OTUs exhibited prominent occurrence and competitive strength in their particular ecological niche. In addition, 1537 shared OTUs (57.2% of their total OTUs) were observed among the CK-treated groups (Fig. 2a), whereas the percentage was merely 39.1% among the N-treated groups (Fig. 2b). These results indicate that there were more different bacterial members among the four temperatures in fertilized soil, which proved once again that N fertilization augmented the bacterial response to increasing temperature.

### Bacterial community composition

**Figure 4:**
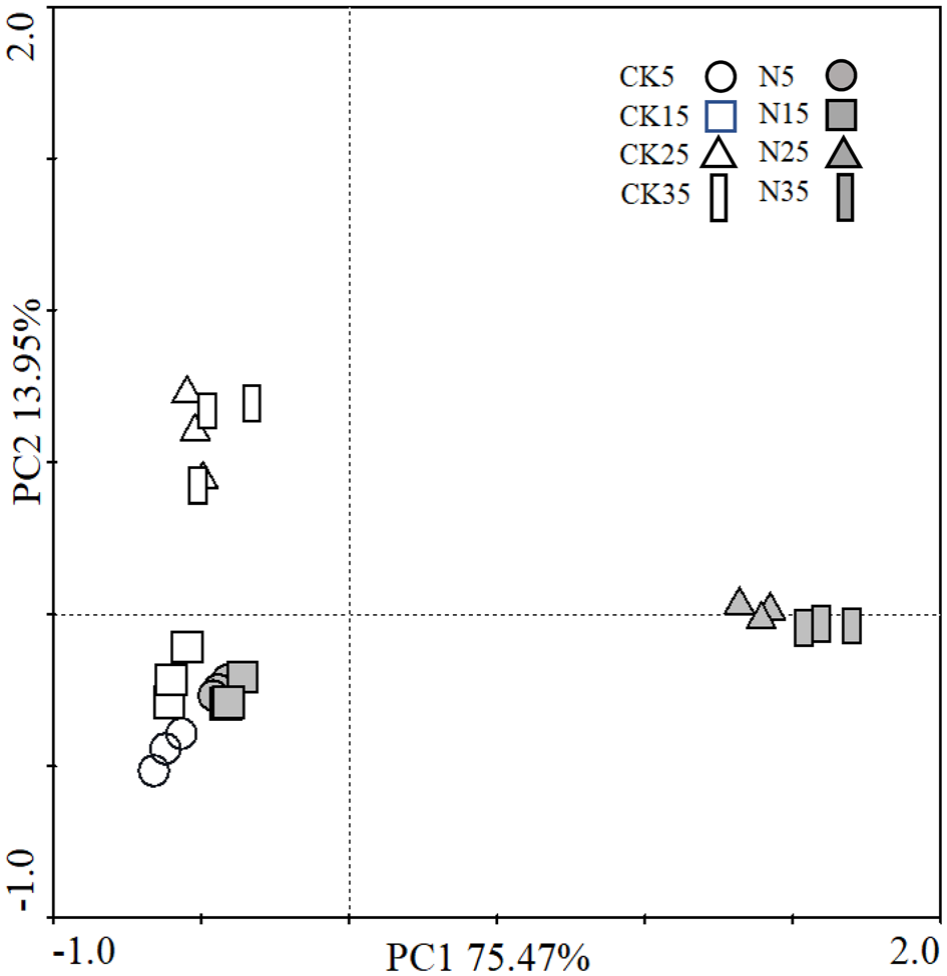
Principal coordinate analysis (PCoA) of the bacterial community structure at different incubation temperatures. Different symbols represent samples under different incubation temperatures. CK, soil samples with no N application; N, soil samples with N application.

PCoA and PERMANOVA were used to quantify and visualize the differences in the bacterial community structure at the OTU level. The first two principal components accounted for 89.4% of the total variability (Fig. 4). Likewise, significant variation occurred only when the incubation temperature was increased to 25 °C and 35 °C. Remarkably, N treatment had a significant effect on the community structure at high temperatures, which was evident by the separation of the CK and N treatments along the PC1 and PC2 axes (R^2^ = 0.87). In contrast, the influence was trivial at low temperatures (R^2^ = 0.44). PERMANOVA also revealed that the shift in the bacterial community structure induced by temperature alone was small (Table S2).

### Microbial composition

**Figure 5:**
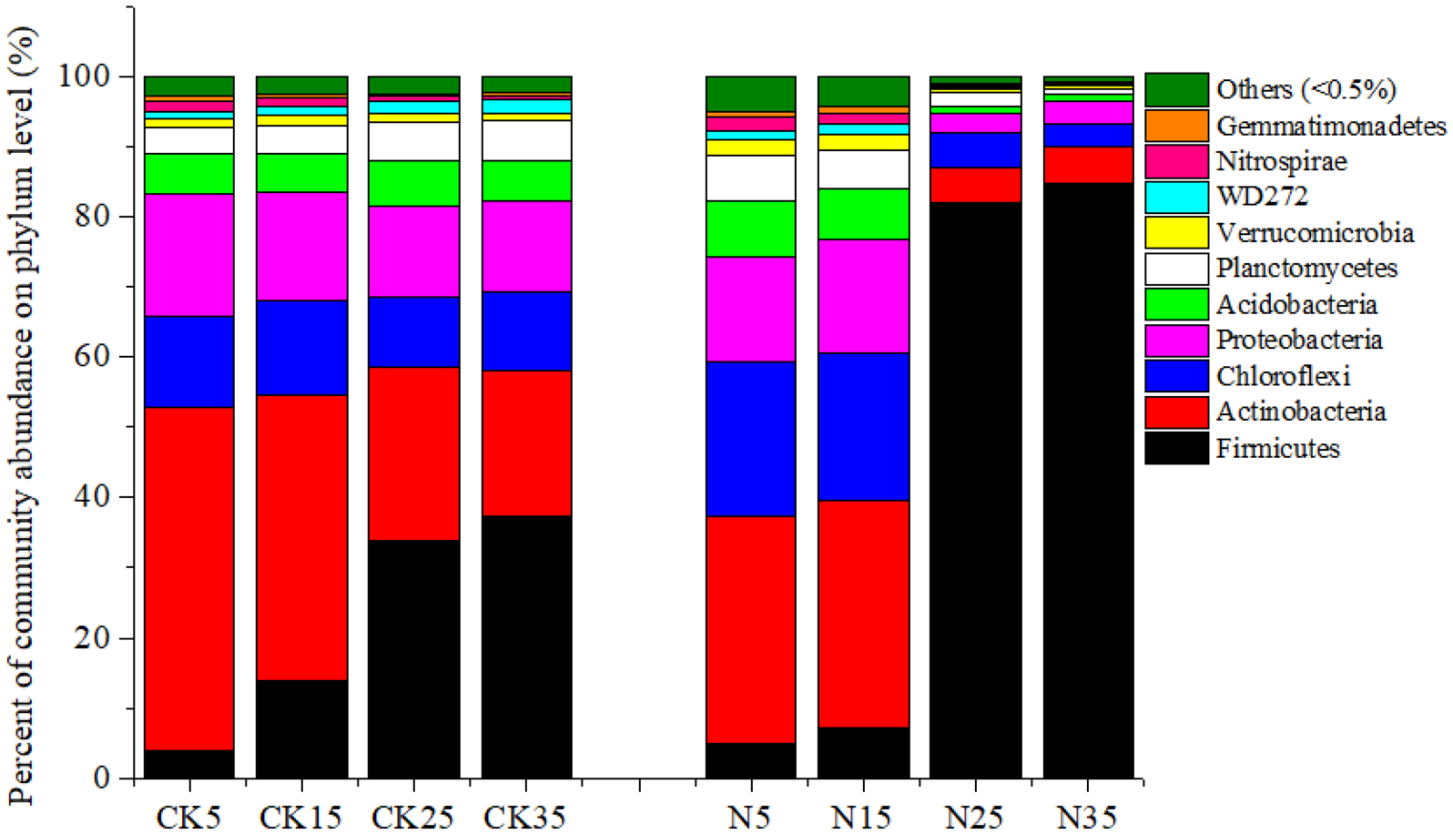
Relative abundance of soil bacteria under different treatments at the phylum level. CK, soil samples with no nitrogen application; N, soil samples with nitrogen application.

Ten phyla were abundant to all treatments (cut-off: > 0.5%) (Fig. 5). *Actinobacteria* was the most abundant phylum at low temperatures, accounting for more than 30% of the total reads. Among this phylum, *Oryzihumus*, *Catenulispora*, and *Kitasatospora* were highly abundant at the genus level (Fig. S1). In addition, *Chloroflexi* and *Proteobacteria* were also highly abundant. When the incubation temperature was at 25 °C and 35 °C, the relative abundance of *Actinobacteria* and *Chloroflexi* significantly decreased, whereas that of *Firmicutes* increased. In particular, N treatment increased the abundance of *Firmicutes* (> 80%), which was the most dominant taxa. *Alicyclobacillus*, *Bacillus*, and *Oxalophagus* belonging to *Firmicutes* were also highly abundant*.* Meanwhile, at the genus level, *Clostridium_sensu_stricto_10* was the most abundant in the non-fertilized treatment.

### Network construction

Co-occurrence patterns have developed into a key assay for ecosystem studies to understand the symbiotic patterns of the microbiome^38^. Thus, we explored how temperature changes and N use affect bacterial co-occurrence (Fig. S2, Table 2). The influence of temperature on the bacterial network was evidently observed in N-fertilized soils and was less evident in CK treatments. A high temperature-induced increased network density and clustering coefficient, lower average path distance and network diameter, and a larger proportion of positive associations (> 80%), compared to those in fertilized, low-temperature soils, indicated that the network was more connected^39^ and complex^27^ with a large proportion of closely related OTUs. The effect of high temperatures on the network of CK treatments was only characterized by a lower network diameter. As for nitrogen-treated soils, significant variation only appeared at high temperatures, with a higher network density and clustering coefficient, as well as lower average path distance and network diameter.

**Table 2:** Node-level topological features of co-occurrence network at different incubation temperatures.

	CK5	CK15	CK25	CK35	N5	N15	N25	N35
Number of nodes	135	143	144	140	176	183	71	68
Number of edges	1836	1720	1778	1597	1817	1873	736	565
Number of positive correlations	1836 (61.1%)	1070 (62.2%)	1077 (60.6%)	933 (58.4%)	979 (53.9%)	1047 (55.9%)	657 (89.3%)	466 (82.5%)
Number of negative correlations	714 (38.9%)	650 (37.8%)	701 (39.4%)	664 (41.6%)	838 (46.1%)	826 (44.1%)	79 (10.7%)	99 (17.5%)
Average degree	27.200	24.056	24.694	22.814	20.648	20.470	20.732	16.618
Network density	0.203	0.169	0.173	0.164	0.118	0.112	0.296	0.248
Average path distance	4.155	3.052	3.488	3.525	4.040	4.110	2.849	1.300
Average clustering coefficient	0.762	0.769	0.749	0.789	0.769	0.756	0.809	0.845
Network diameter	19	10	8	7	13	14	7	3

## Discussion

Temperature is a key determinant of microbial growth^9^. Our results confirmed that temperature significantly affects the soil bacterial community in many aspects, including bacterial abundance, α-diversity, community structure, taxonomic composition, and interspecies interactions. Here, bacterial diversity and interspecies interactions to temperature were of particular interest because they are relevant ecological functions. Our results showed that warming induced lower soil bacterial α-diversity, consistent with the results of other studies^9,11,29^. A previous study has shown that high temperatures may inhibit many indigenous organisms, allowing the colonization of other thermally-adapted microbes^40^. Furthermore, we observed that many psychrophilic taxa were diminished under high temperatures, and their niche breadths and relative abundance were very low. These observations suggest that the species occupying narrow thermal niches with low abundance are likely to be more vulnerable to global warming^41^. In contrast, several mesophilic OTUs were highly abundant, most of which were belonged to *Firmicutes*. The abundance of *Firmicutes* significantly increased under warming, even in the fertilized soils. *Firmicutes* is dominant in tropical soils^42^ and are presumed to possess better thermal adaptability and water preference^43^. *Firmicutes* feed on the necrotic cells from dead indigenous taxa^44^ and then grow rapidly, leading to high abundance and resulting in low α-diversity^44^. However, low temperature is a major environmental stress for many bacteria^45^, allowing for a lower competitive pressure that promotes higher α-diversity.

The decrease in bacterial diversity under warming may damage certain ecosystem functions^5,6^, especially those in extensively human-disturbed ecosystems^46^. We aimed to identify the strategy of microbial communities for compensating the impaired functions. A previous study revealed that frequent and close interspecies interactions could improve various ecosystem functions^21^; hence, we generated a network indicating microbial interactions. We found that warming enhanced network connectivity and complexity. Moreover, the lowest microbial diversity coexisted with the most complex and closest association in fertilized soils under high temperatures. Therefore, microbes may have exhibited improved cooperation to maintain the impaired ecosystem functions^27,47^. These results concurred with those of previous studies. Yuan et al.^27^ reported that climate warming enhanced the microbial network complexity of grassland soil. Zhao et al.^25^ found that lower microbial diversity was associated with more complex and closer interactions in subtropical and tropical forest soils. Furthermore, a large proportion of positive associations was observed in soils in high temperature and N treatment, which also improved ecosystem functions^48^. In addition, they both showed minor variation in non-fertilized soils, thus, they changed perfectly in step. Although Ratzke et al.^23^ verified the mechanistic link between them using culture experiments, we first found, by performing incubation experiments, that the two changed at an approximately constant pace regardless of the extent of change. Increasing evidence suggests that microbes exhibit reshaped structure under different environments to achieve ecosystem functions^6,49^. Therefore, we speculated that intrinsic relationships, that is, complementary functions, are present between microbial network complexity and diversity. However, we cannot verify these speculations because the specific microbial function was not measured. Thus, further studies focusing on certain ecosystem functions and related microbes are necessary to validate our presumption.

In addition, we found that the soil bacterial community did not change continuously with increasing temperature; it exhibited a two-phase pattern. Bacterial communities under low-temperature treatments (5–15 °C) were similar, and those under high-temperature treatments (25–35 °C) were also similar, suggesting that bacteria possess temperature adaptability to some extent. Several studies have also reported that soil bacterial diversity, composition, and structure are stable when the temperature variation is relatively low^50,51^. Finlay and Cooper^52^ suggested that the composition of microbial communities often changed when environmental factors vary in high amplitudes or frequencies. However, bacterial adaptation to temperature was observed to be finite. As incubation temperature increased from 15 to 25 °C, each feature of the soil bacterial community in this study dramatically changed, implying that a threshold effect of the temperature response might exist. Previous studies on soil nitrogen-transforming bacteria found that the most striking temperature response appeared at low-to-moderate temperatures^53^, which was the same with 15 °C and 25 °C in this study. It seemed that the common rule was applied to total bacteria as well. The paddy soil investigated in the present study is located in a region with a subtropical monsoon climate and a mean annual temperature of approximately 15–20 °C, which is sensitive temperature range. Thus, we speculated that future global temperature fluctuations would profoundly change the soil microbes in this region, which might explain the higher temperature sensitivity of this region^54^.

Nitrogen availability has been recognized as a key factor influencing the response of the soil microbial community to temperature^33–35^. Our study suggests that the temperature range is a probable reason for the inconsistent results. In high-temperature treatments, nitrogen exerted a strong negative effect on bacterial α-diversity. Positive effects, such as an increase in biodiversity, occurred with low nitrogen; however, the effect became negative when the amount of N added exceeded a threshold^30^. In the present study, nitrogen application occurred at a rate of up to 200 kg N ha^−1^, probably exceeding the threshold, and thus had a negative influence on bacterial diversity. However, nitrogen-fertilized soils had more complex and closer networks than CK-treated soils at high temperatures, indicating that an ecosystem with more available resources could maintain a complex structure^31^. Furthermore, adequate resources can sustain the growth of numerous soil microbes, resulting in higher bacterial abundance. These microbes coexist with diverse types of associations, including mutualism, competition, and predation^18,55^, leading to more complex networks. Nitrogen application enhanced diversity loss and microbial interaction under high temperatures, as warming could select for fast-growing bacteria^27^. Fast-growing taxa possess high resource acquisition rates^56^, thereby responding quickly to nitrogen treatment. In contrast, the impact of nitrogen on the bacterial community was insignificant in low-temperature treatments. A lag phase during bacterial growth is necessary to adapt to the new environment, which was longer at low temperatures^45,57^. However, the incubation times used in this study were presumably not long enough to allow soil bacteria to respond to nitrogen addition at low temperatures, considering that soil bacteria have a mean generation time of days at 20°C^58^. Therefore, we concluded that nitrogen treatment increased the soil microbial community response to different temperatures mainly by enhancing the response of the bacterial community to high temperatures. Owing to limited nitrogen treatments (non-fertilized and 200 kg N ha^−1^) in this study, our study reported preliminary results examined using setting nitrogen gradients. In future studies, we can target the optimum nitrogen quantity to retain microbial diversity, activate microbial interactions, and ultimately understand the ecological function.

## Conclusion

Temperature strongly affected soil microbial distribution. Elevated temperature significantly decreased the bacterial α-diversity, altered the community composition, and increased the network complexity and connectivity. However, the variation did not occur continuously with increasing temperatures, which was similar under low-temperature treatments (5–15 °C), as well as within high-temperature treatments (25–35 °C), respectively. Moreover, N treatment strengthened their variance. The lowest bacterial diversity and the most complex and closest network coexisted at higher temperatures in N-fertilized soils. Overall, we concluded that the combination of warming and N treatment strongly changed soil bacterial diversity and interactions, and decreased bacterial diversity led to close microbial interactions, which is important for maintaining ecosystem functions and microbial biodiversity loss. Further research is still needed to investigate the relationship between bacterial diversity and interactions in various ecosystems and under different environmental stresses to illustrate compensation mechanisms.

## Experimental methods

### Soil sample collection and processing

Typical acidic paddy soils, developed from quaternary red clay, were collected from the Taoyuan Agroecosystem Research Station (111°269E–28°559 N; altitude, 92.2–125.3 m) in Hunan Province, China, in October 2014. Detailed soil collection methods, climate factors, and major soil properties have been reported previously^59^.

### Soil community incubation

Soil samples were preconditioned in the dark with 25% water-filled porespace (WFPS) at 25 °C for 4 d before the start of the experiment. Briefly, 200 g (dry weight) of soil was placed into a 1000 ml glass bottle. NH_4_NO_3_ was added to half the volume at 720 µg N g^−1^ of dried soil, which is equivalent to 200 kg N ha^−1^ when added to 200 g of soil with a surface area of 72 cm^2^, whereas the other half was not fertilized (CK). The moisture content of the soil was adjusted to 125% WFPS. Subsequently, the bottles were uncapped and incubated at 5 °C, 15 °C, 25 °C, and 35 °C for 96 h. Three independent replicates were prepared for each temperature. Notably, we defined incubation at 5 °C and 15 °C as low-temperature treatments, and while that at 25 °C and 35 °C as high-temperature treatments. After incubation, the soil was destructively sampled, and approximately 20 g was stored at − 80 °C until molecular analyses.

### DNA extraction, qPCR, and high-throughput sequencing

Microbial genomic DNA was extracted from soil (0.5 g) after 96 h of incubation, as described by Chen et al.^60^ with minor modifications. The MP FastPrep®-24 (MP Biomedicals, LLC, Santa Ana, CA, USA) was used in the first step instead of the 1-h water bath treatment. DNA quality was observed using 1% agarose gel electrophoresis the NanoDrop NA-1000 spectrophotometer (Nanodrop Technologies, Wilmington, DE, USA). Triplicate extractions were performed for each incubation, and the resulting DNA was pooled and stored at − 80 °C until analysis. Real-time quantitative polymerase chain reaction (q-PCR) was performed to amplify soil genomic DNA using the primer set 1369F (5′-CGGTGAATACGTTCYCGG-3′) and 1492 R (5′-GGWTACCTTGTTACGACT-3′). The 16S rRNA gene was amplified using the primer set 338F (5′-ACTCCTACGGGAGGCAGCAG-3′) and 806R (5′-GGACTACHVGGGTWTCTAAT-3′) for high-throughput sequencing. PCR reactions and thermal programs are listed in Table S3. High-throughput sequencing was performed using the Illumina PE300 platform at Majorbio Bio-pharm Technology Co., Ltd. (Shanghai, China). Sequence analysis was performed using Mothur software (version v.1.30.2). The sequences were denoised and checked for chimeras and then clustered based on 97% identity. A total of 581,644 sequence reads and 2794 OTUs were obtained.

### Network analysis

Co-occurrence network analyses were performed to explore how elevated temperature and nitrogen treatment affect the co-occurrence patterns of bacterial microbial communities. To simplify the network for better visualization, OTUs with an average relative abundance < 0.1% in each group were excluded^61^. Interaction networks were constructed using CoNet v1.1.1 in Cytoscape v.3.6.1 based on the Pearson and Spearman correlation values, mutual information similarity, and Bray–Curtis and Kullback–Leibler dissimilarity measures. All networks were visualized using the Fruchterman–Reingold layout with 9999 permutations and implemented in Gephi^62^. The topological features of each sub-network were calculated, including the total number of network nodes, total number of edges, average degree, network density, network diameter, average path distance, and average clustering coefficient.

### Statistical analysis

Analysis of variance (ANOVA) was performed to examine the effects of incubation temperature and nitrogen application on the abundance and diversity of soil bacterial community using SPSS 18.0. A Venn diagram was generated to show the overlapping OTUs between the different treatments. The “niche breadth” approach^63^ was used to quantify the habitat specialization of each OTU. The formula is as follows:

$${B}_{j}=\frac{1}{\sum_{i=1}^{N}{{P}_{ij}}^{2}}$$

where B_j_ is the niche breadth, P_ij_ is the proportion of OTU j at given site i, and N is the total number of sites. Principal coordinate analysis (PCoA) was conducted to determine bacterial distribution profiles. Permutational multivariate analysis of variance (PERMANOVA) based on the Bray–Curtis dissimilarities of OTUs was used to measure the effects of temperature, fertilization, and their interactions on bacterial community composition.

## Supplementary Information

Supplementary Information.

## Data Availability

All *16S* rRNA gene sequences have been deposited in the GenBank Sequence Read Archive under the accession number PRJNA762206.
