# Supplementary material for: Nitrogen use aggravates bacterial diversity and network complexity responses to temperature
Source: Sci Rep. 2022 Aug 17;12:13989. doi: 10.1038/s41598-022-15536-5 (PMC9385738; doi:10.1038/s41598-022-15536-5)

Table S1 Two-way ANOVA showing the effects of temperature and fertilization on the soil total bacterial α-diversity

| Alpha diversity index | Treatment | Type III Sum of Squares | df | Mean Square | F | Sig. |
| --- | --- | --- | --- | --- | --- | --- |
| Ace | temperature | 616433.143 | 3 | 205477.714 | 51.353 | 0.000 |
|  | fertilization | 2095.072 | 1 | 2095.072 | .524 | 0.480 |
|  | temperature * fertilization | 67752.187 | 3 | 22584.062 | 5.644 | 0.008 |
| Chao1 | temperature | 1687944.737 | 3 | 562648.246 | 124.398 | 0.000 |
|  | fertilization | 218149.876 | 1 | 218149.876 | 48.232 | 0.000 |
|  | temperature * fertilization | 604643.516 | 3 | 201547.839 | 44.561 | 0.000 |
| Shannon | temperature | 12.149 | 3 | 4.050 | 190.224 | 0.000 |
|  | fertilization | 2.384 | 1 | 2.384 | 111.961 | 0.000 |
|  | temperature * fertilization | 11.819 | 3 | 3.940 | 185.049 | 0.000 |
| Simpson | temperature | .020 | 3 | .007 | 76.226 | 0.000 |
|  | fertilization | .011 | 1 | .011 | 126.397 | 0.000 |
|  | temperature * fertilization | .028 | 3 | .009 | 106.056 | 0.000 |
| PD | temperature | 8767.455 | 3 | 2922.485 | 125.776 | 0.000 |
|  | fertilization | 196.387 | 1 | 196.387 | 8.452 | 0.010 |
|  | temperature * fertilization | 3741.614 | 3 | 1247.205 | 53.676 | 0.000 |

Table S2 PERMANOVA representing the influence of temperature and fertilization on soil bacterial distribution

Table S3. PCR reactions and thermal profiles for PCR amplification of 16S

| Primer | Primer sequence (5′- 3′) | PCR reactions | Thermal profile | Fragment length/ bp |
| --- | --- | --- | --- | --- |
| 1369F  1492R | CGGTGAATACGTTCYCGG | 5 ng template DNA, 5 μL 2×SYBR Premix Ex TaqTM, 0.5 μL (10 μM) of both forward and reverse primers, and deionized water to a total of 10 μL | 95°C 3 min; 30 cycles of 95°C 45 s, 54°C 45 s, 72°C 60 s; 72°C 10 min | 123 |
|  | GGWTACCTTGTTACGACT |  |  |  |
| 338F  806R | ACTCCTACGGGAGGCAGCAG | 10 ng template DNA, 4 μL 5×FasrPfu buffer, 2.5 μl mMdNTPs, 0.4 μl FastPfu polymerase, 0.4 μL (5 μM) of both forward and reverse primers, and deionized water to a total of 20 μL | 95°C 3 min; 35 cycles of 95°C 30 s, 55°C 30 s, 72°C 45 s; 72°C 10 min | 468 |
|  | GGACTACHVGGGTWTCTAAT |  |  |  |

Figure S1 Taxonomy information of bacteria at genus level under different incubation temperatures. CK, soil samples with no nitrogen application; N, soil samples with nitrogen application.


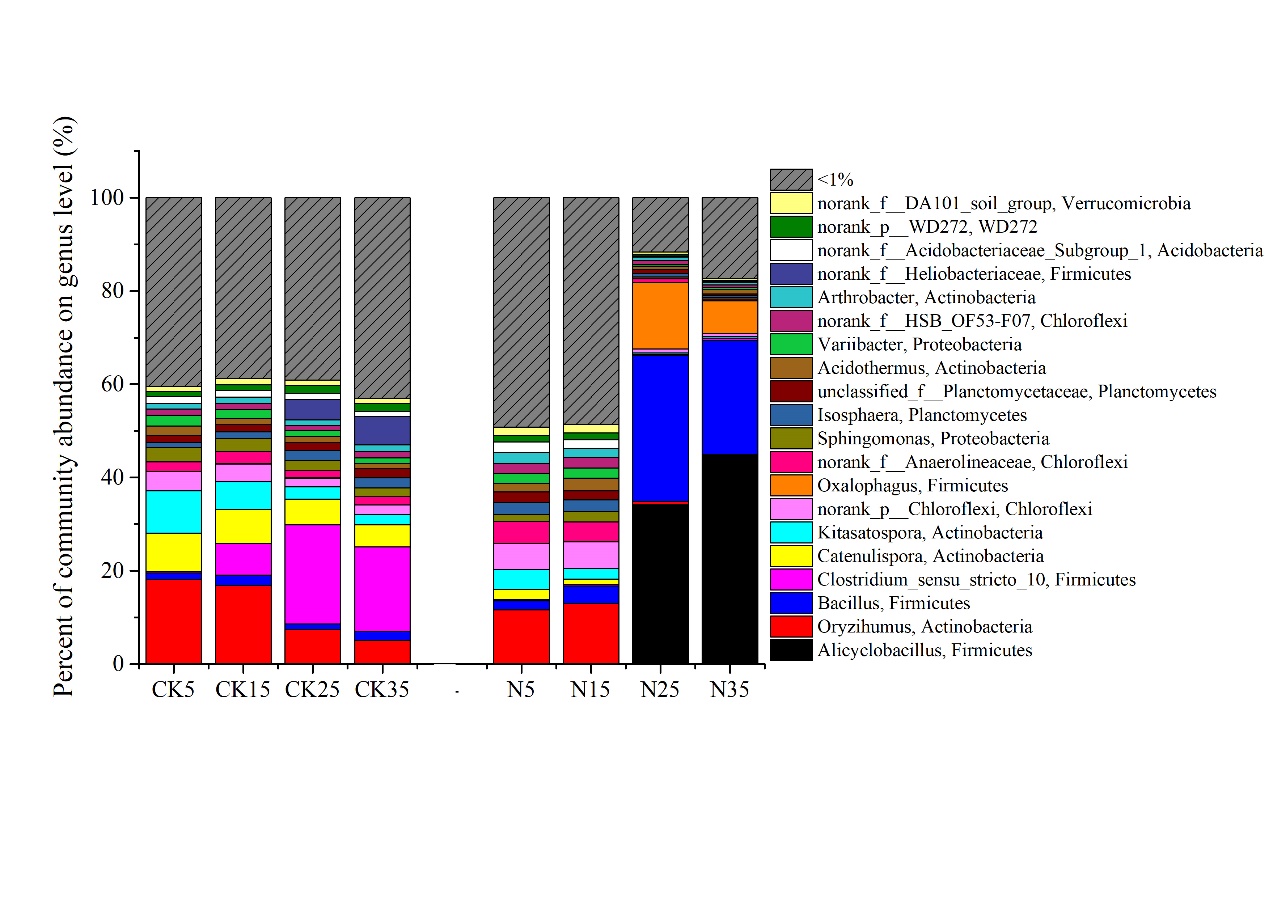


Figure S2 The co-occurance network of bacterial communities under CK and N treatments across each temperature. Soil samples with no nitrogen application under 5 ℃ (a), 15 ℃ (b), 25 ℃ (c), 35 ℃ (d), soil samples with nitrogen application under 5 ℃ (e), 15 ℃ (f), 25 ℃ (g), 35 ℃ (h).


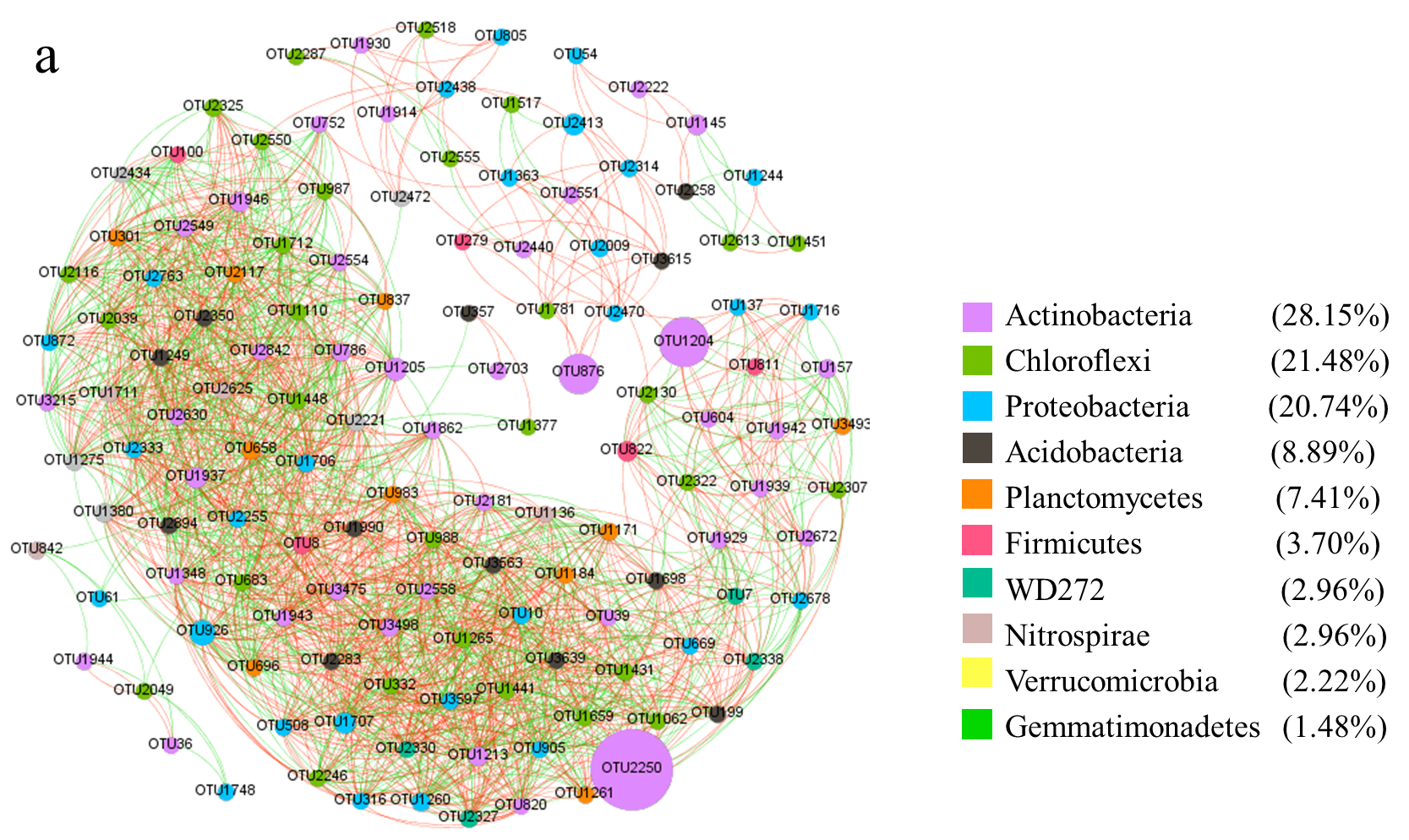


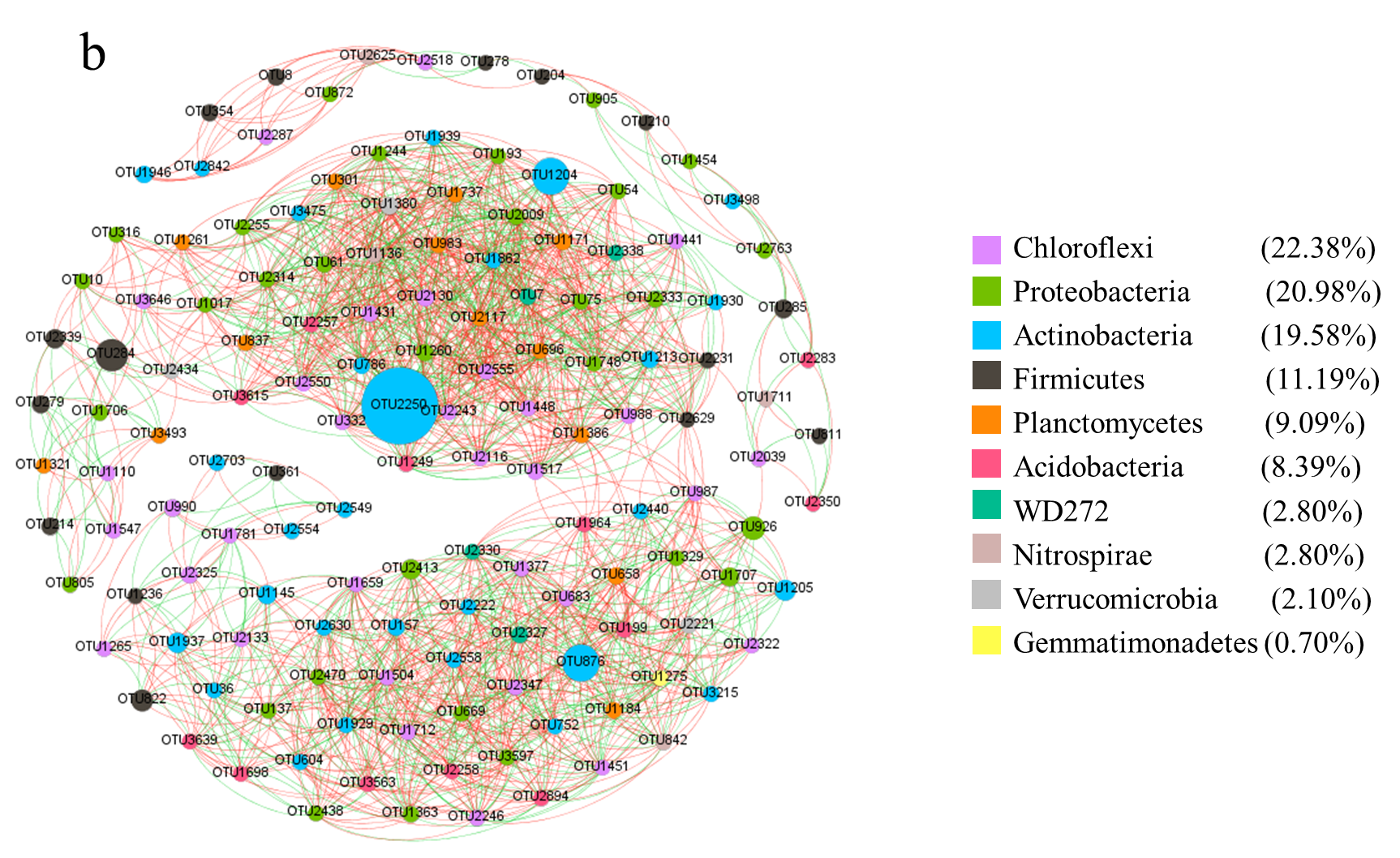


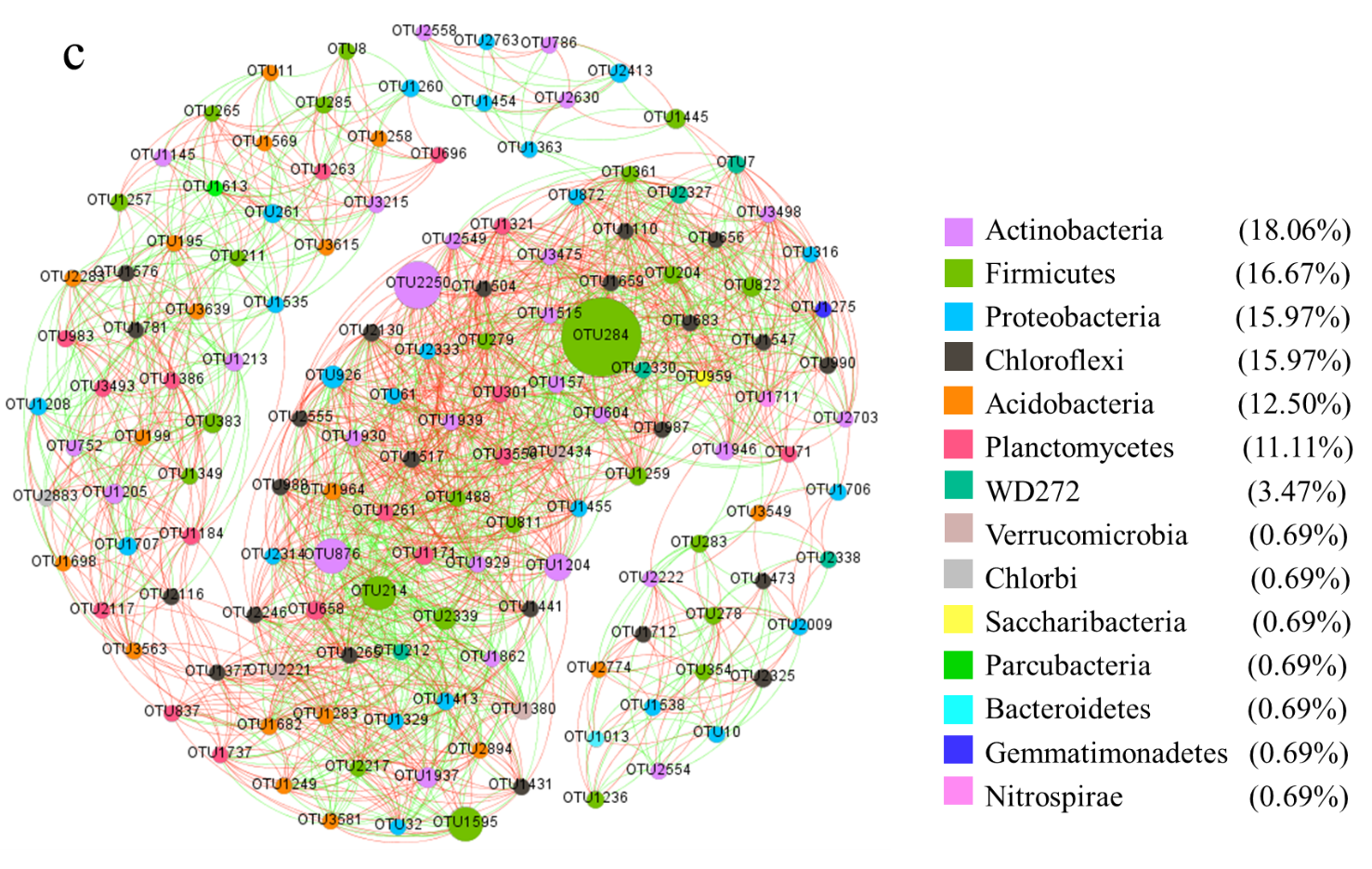


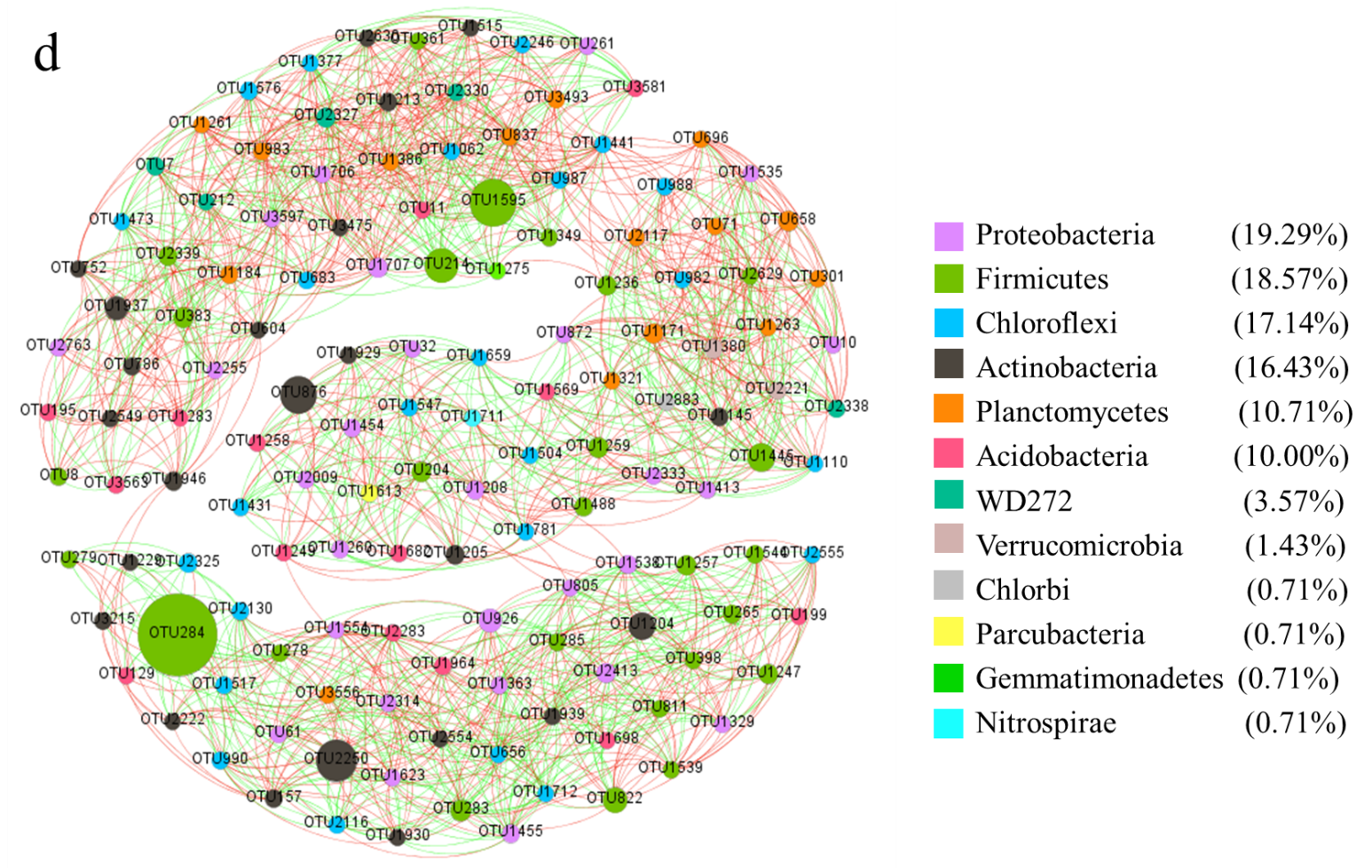


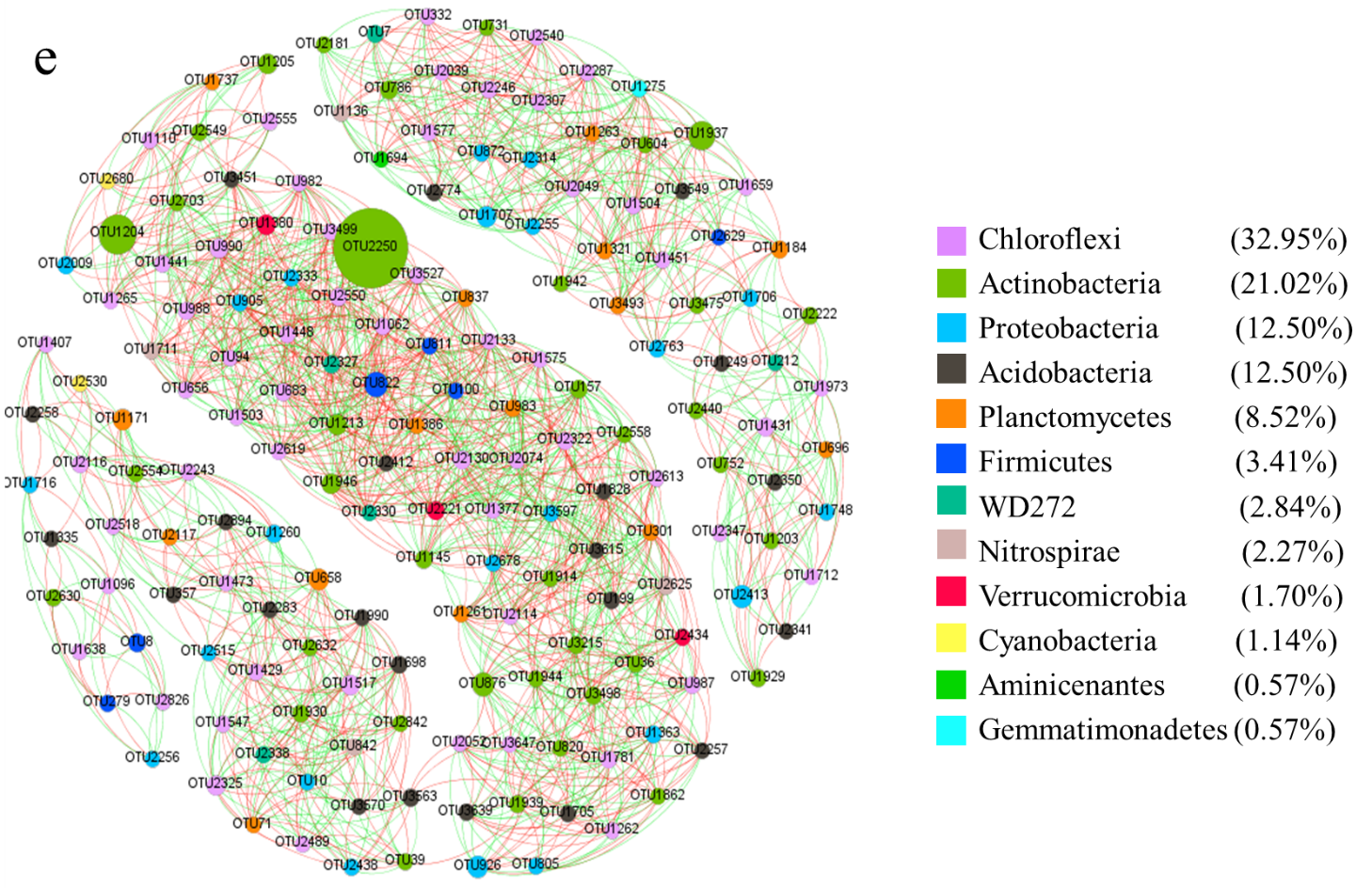


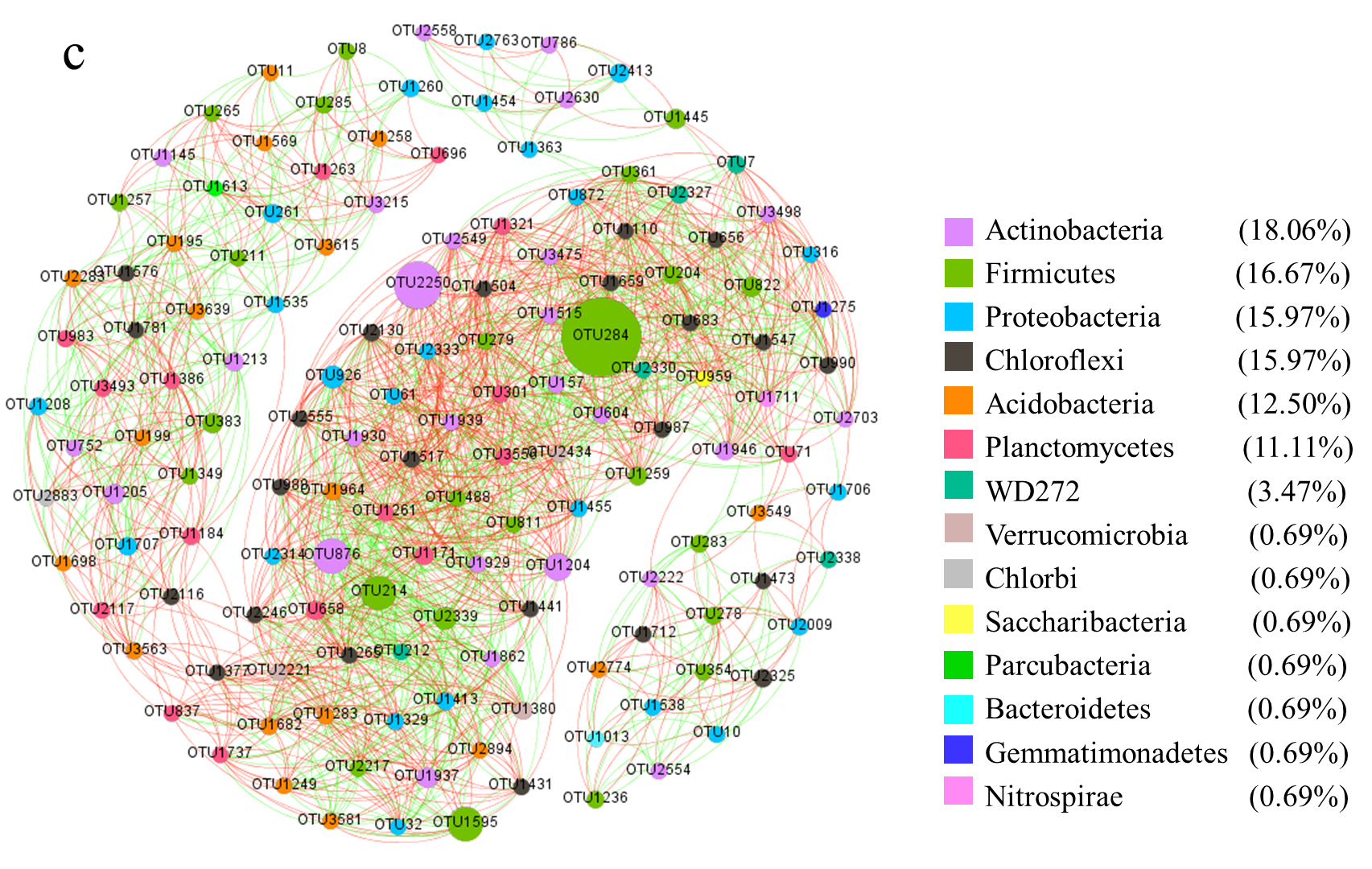


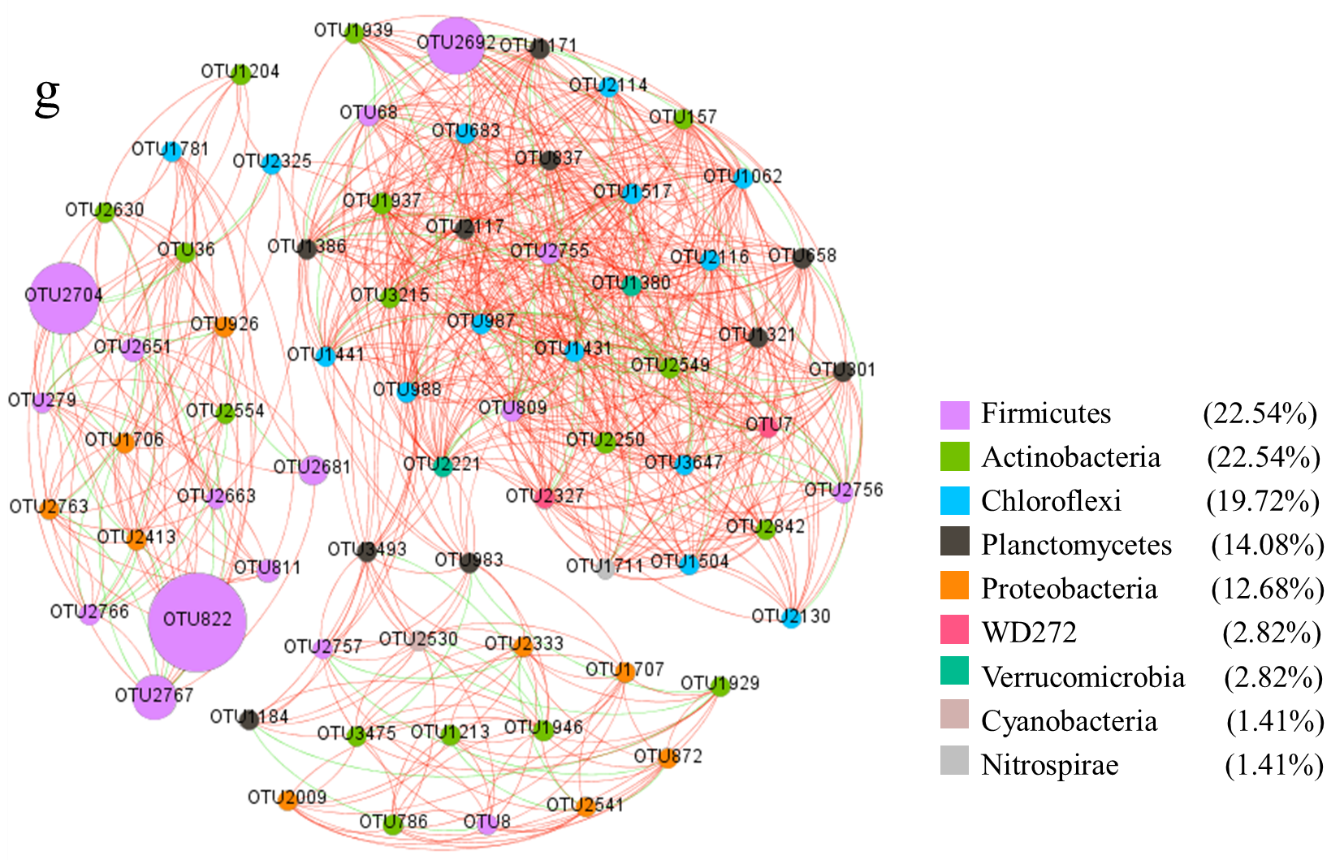

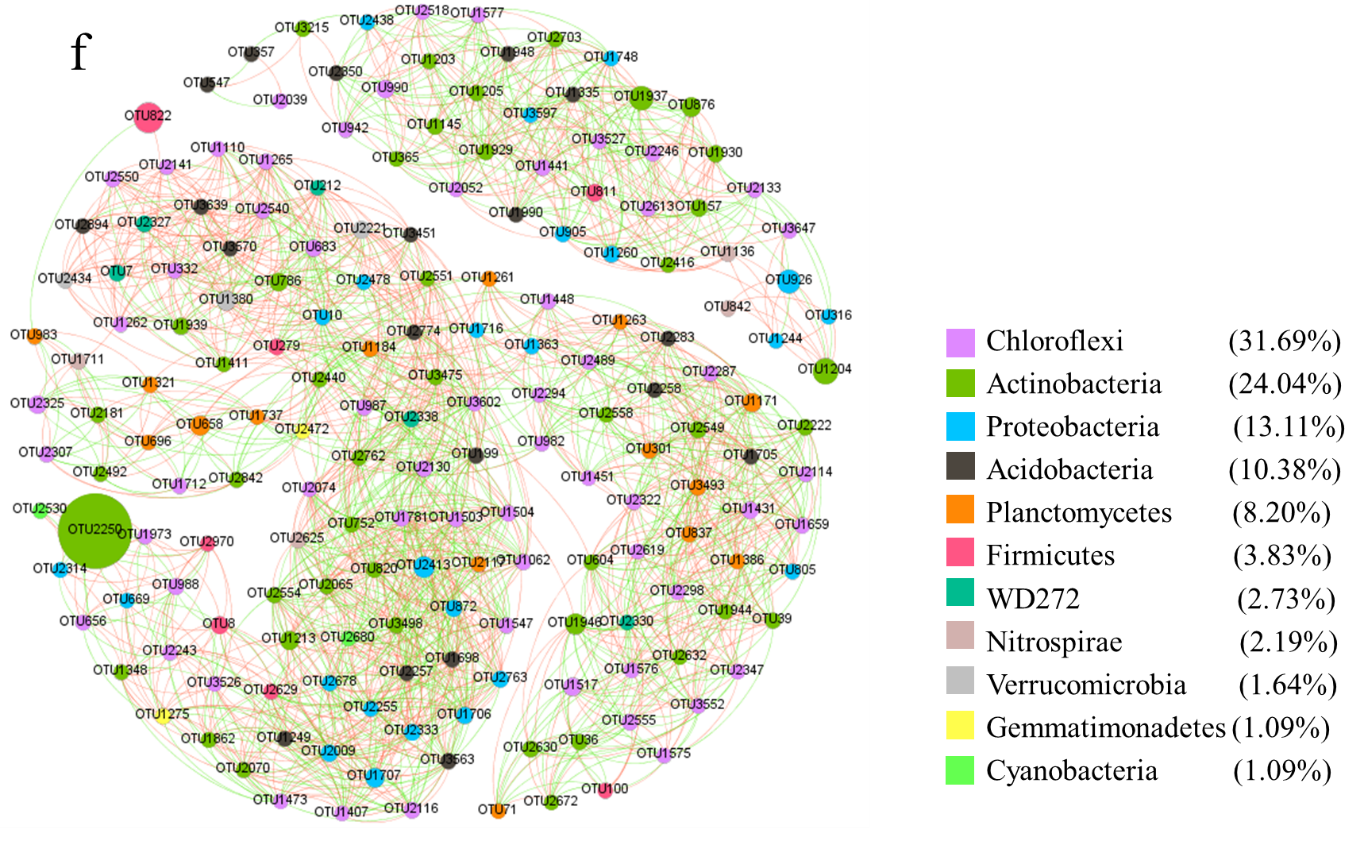


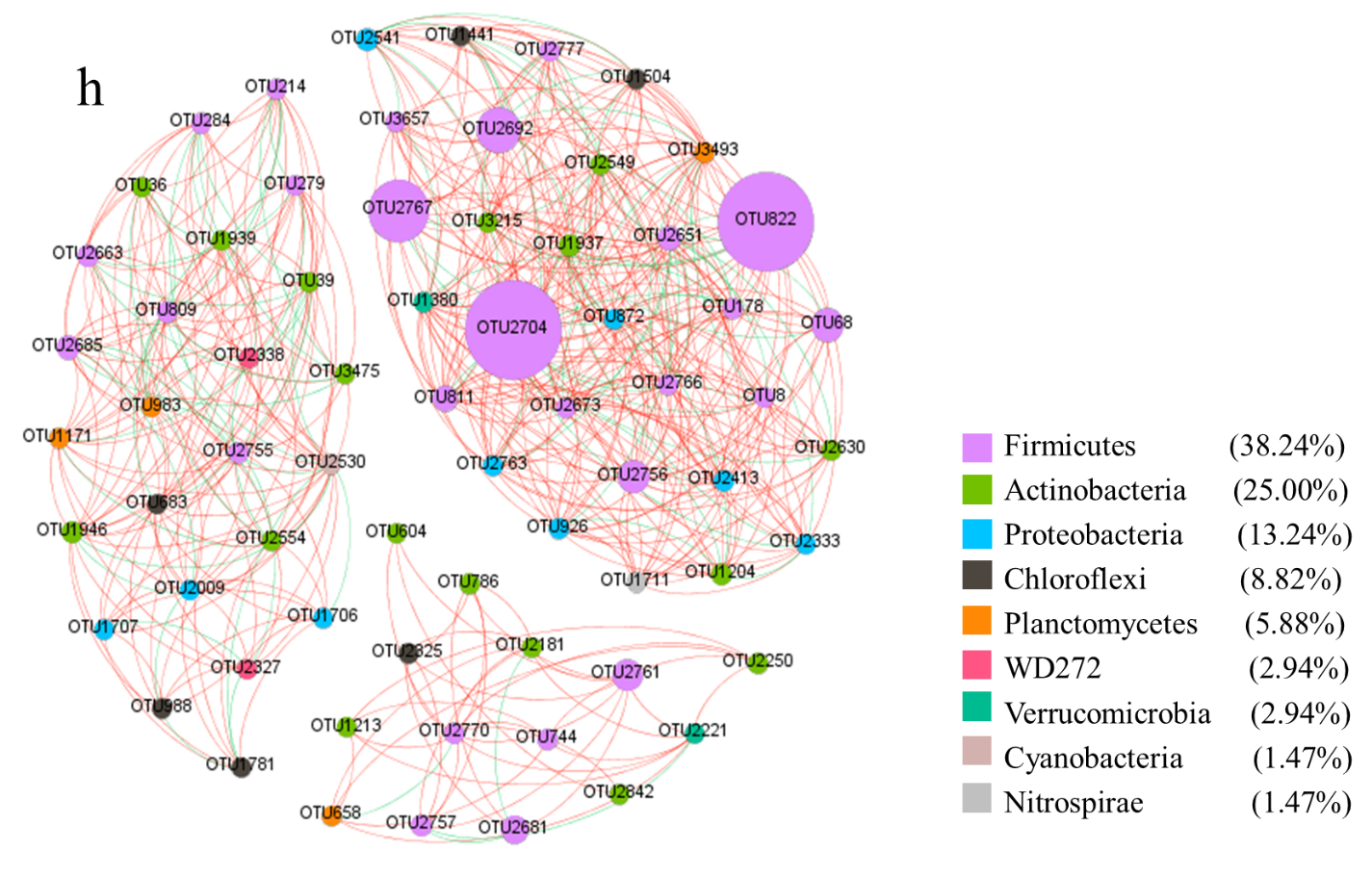

Supplement: Supplementary file 1 — Supplementary Information. [file 41598_2022_15536_MOESM1_ESM.docx]
